# A Prospective Observational Clinical Cohort of Women with Suspected Breast Implant Illness

**DOI:** 10.3390/jcm13154394

**Published:** 2024-07-27

**Authors:** Karlinde A. Spit, Siham Azahaf, Christel J. M. de Blok, Prabath W. B. Nanayakkara

**Affiliations:** Section General Internal Medicine, Department of Internal Medicine, Amsterdam Public Health Research Institute, Amsterdam University Medical Centres, Location VUmc, 1081 HZ Amsterdam, The Netherlands

**Keywords:** silicone breast implants, breast implant illness, implant removal, explantation

## Abstract

**Background**: This study aims to describe a prospective clinical cohort of patients with silicone breast implants and suspected Breast Implant Illness (BII). **Methods**: Women were included in a specialized silicone outpatient clinic at Amsterdam UMC, the Netherlands. Baseline characteristics were collected including medical history, implant details, and symptoms. Experienced physicians categorized BII suspicion as high, moderate, or low, based on symptoms and after exclusion of other probable causes. Additionally, participants completed questionnaires assessing symptoms and daily life impact at baseline, 6 months, 1, 2, and 5 years. For this initial analysis, the results from the first three years of inclusions were collected. **Results**: Data from 353 women were collected from December 2020–December 2023. At baseline, the most reported symptoms were fatigue, arthralgia, myalgia, and morning stiffness, accompanied by local symptoms in 83.3% of patients. At the clinic, 112 women were categorized as having high suspicion of BII, 147 women as moderate, and 94 women as low. At follow-up, 182 women (51.6%) opted for explantation. Following explantation, women with a high or moderate suspicion of BII experienced more significant symptom improvement, accompanied by a decrease in anxiety and a greater sense of control over their illness, compared to women with a low suspicion of BII. **Conclusions**: Our study highlights a distinct interplay of systemic and local symptoms among women with suspicion of BII. Women with a high or moderate BII suspicion benefit significantly more from explantation than women with low suspicion. Experienced physicians are pivotal in effectively assessing and guiding this patient group, highlighting the need for tailored clinical approaches.

## 1. Introduction

Silicone breast implants are commonly used medical devices for breast augmentation and reconstruction. Despite the widespread acceptance and popularity of this procedure, concern about potential health implications has grown over the years [1,2,3,4]. Some women with breast implants experience the development of a constellation of systemic symptoms, attributed to their breast implants [5,6,7,8,9]. These include but are not limited to, fatigue, morning stiffness, arthralgia, myalgia, and night sweats, often accompanied by local symptoms such as breast pain or axillary lymphadenopathy [6,9]. Multiple cohort studies and case series have documented improvement of these symptoms in the majority of patients after the removal of their implants [10,11]. While no universally accepted term for this constellation of symptoms exists, many women with breast implants, and some within the medical community, currently refer to it as ‘Breast Implant Illness (BII)’.

However, the etiology and pathophysiology of BII remain elusive, contributing to the controversy and challenges in understanding this phenomenon. The most generally accepted hypothesis states that silicone implants incite an immune response in susceptible individuals. The immune system’s recognition of silicone as a foreign substance may lead to chronic inflammation, potentially giving rise to the broad range of reported symptoms [4,12,13].

Recently, the concept of silicone migration has gained more attention in this regard. While modern implants were designed to be durable and resilient, rupture rates are still of concern. The prevalence of implant ruptures ranges from 10–15% after breast augmentation to 20–25% after breast reconstruction within a decade of implantation, even with modern implants [14,15,16,17] Silicone particles are released into the surrounding tissues and spread mainly through the lymphatic system, where silicone accumulation is most commonly observed in axillary lymph nodes [18]. These distant silicone particles might act as triggers for inflammatory responses, extending beyond the local site of implantation [13,19]. Notably, silicone migration can occur even in the absence of implant rupture, through a process known as ‘gel bleed’ [18,20].

In contrast, some argue that BII is mainly a ‘social media phenomenon’. This perspective suggests that social media platforms generate or contribute to anxiety and self-diagnosis [21] The displaying of (nonscientific) information about BII on various social media platforms results in some physicians refusing to acknowledge its existence, or automatically associating BII symptoms with psychological factors or merely a nocebo effect [22].

This ongoing controversy and uncertainty raises great concern among women with breast implants. A growing number of women consult medical specialists, such as plastic surgeons, internists, and rheumatologists, seeking answers regarding a potential association between their symptoms and breast implants [23]. Currently, no international guidelines are available for this specific patient group, leading to frustration among both women with breast implants and the medical specialists they consult, some of whom feel ill-equipped to provide adequate medical support. Many argue that the symptoms, such as fatigue and arthralgia, are nonspecific and therefore not distinctive for BII. Patients, in turn, may be fearful of being labeled as having an illegitimate disease [21]. Recognizing this gap between patients and the medical field underscores the pressing need for more awareness and clarity regarding this phenomenon through research.

Two years ago, we reported the findings of a retrospective cohort study from our specialized silicone outpatient clinic at Amsterdam UMC, VUmc location, in the Netherlands [9]. At this clinic, women suspected of having BII were evaluated by a team of experienced physicians. BII was suspected when patients present with typical symptoms, and after excluding alternative diagnoses by performing an extensive clinical assessment using medical history, physical examination, laboratory, and radiological examinations. Our retrospective cohort study demonstrated that the majority of women with suspected BII who chose to undergo implant removal experienced a notable improvement in their symptoms [9]. Moreover, this improvement in their symptoms was significantly more pronounced in comparison to the group of women with a suspicion of BII who opted not to undergo explantation. However, the retrospective nature of this cohort study had certain limitations.

Hence, we present a prospective observational clinical cohort study conducted at our silicone outpatient clinic. The objective of our current study is to elucidate the patient population and their presenting symptom patterns, outline our diagnostic trajectory, evaluate post-implant removal outcomes, and assess the impact on daily life and psychological well-being. We hereby aim to contribute to improving patient outcomes and bridge the gap between patients and medical professionals.

## 2. Patients and Methods

Women visiting the specialized silicone outpatient clinic at Amsterdam UMC, location VUmc were invited to participate in this study starting from December 2020. In December 2023, the results from the first three years were collected and analyzed. This initial analysis focuses on baseline symptom patterns, first post-removal outcomes, and the impact on daily life. As the study continues, future analyses will address long-term post-removal outcomes and the development of symptoms over time.

This study was reviewed by the Ethical Review Board of the Amsterdam UMC, VU University Medical Centre Amsterdam (reference number: 2020.501). It was determined that the Medical Research Involving Human Subjects Act (WMO) does not apply to this study. Written informed consent was obtained from all participants.

### 2.1. Clinic Visit

The English version of the comprehensive clinical protocol is provided as Supplementary Material (Supplementary S1; Guideline). During the first outpatient clinic visit, an experienced consultant physician conducted an extensive assessment of the patients’ medical history, implant history and surgical details, and systemic and local symptoms. A physical examination was performed, with attention to the breasts and axillary region. Blood tests were conducted to rule out alternative explanations for their symptoms, which included erythrocyte sedimentation rate (ESR), C-reactive protein (CRP), hemoglobin (Hb), thrombocytes, leucocytes with differentiation, liver enzymes, renal function, thyroid stimulating hormone (TSH), anti-nuclear antibodies (ANA), vitamin D (1,25-OH), ferritin, and vitamin B12 concentration. Additional tests, such as the presence of anti-cyclic citrullinated peptide (anti-CCP) or rheumatoid factor (RF), or other similar tests, were performed on the indication. Radiological examinations such as ultrasound and/or breast MRI scans were performed when indicated.

The medical history, medication use, allergies, and intoxications of participating patients were documented by the treating medical physician and stored within a secure electronic data capture database (Castor EDC). Clinical suspicion of BII for each patient was categorized and documented as ‘high suspicion’, ‘moderate suspicion’, or ‘low suspicion’. A ‘high suspicion’ classification was based on the presence of multiple typical systemic symptoms such as fatigue, arthralgia, myalgia, morning stiffness, and night sweats, along with local symptoms such as breast pain or axillary lymphadenopathy, and the exclusion of other plausible causes for these systemic symptoms. Patients were categorized as ‘moderate suspicion’ if they exhibited an incomplete typical systemic symptom pattern, lacked local symptoms, or if there was a plausible alternative cause for some of the symptoms. A ‘low suspicion’ was assigned when there was a clear alternative cause that could fully account for the symptoms, or when the symptom pattern was evidently atypical. Patients with a high suspicion were advised to undergo implant removal. For those with a moderate suspicion, recommendations were made on an individual basis, using shared decision-making principles. For patients with a low suspicion of BII, it was discussed that implant removal in their case would most likely not result in symptom improvement. However, it is important to note that the ultimate decision regarding implant removal was left to the discretion of each patient across all groups.

### 2.2. Surveys

Digital surveys were sent to all patients within the week following their first clinic visit, comprising a questionnaire including their implant history and details, the presence and severity of both local and systemic symptoms, how the symptoms impacted their daily lives, and a brief illness perception questionnaire (BIPQ) [24]. A cross-cultural adapted Dutch version of the BIQP was used for this study (IPK-Q), designed to rapidly assess the cognitive and emotional representations of their illness. The comprehensive questionnaire was scheduled for repetition at 6 months after their initial clinic visit, and subsequently at 1, 2, and 5 years. The questionnaires also included various free-text responses.

### 2.3. Statistical Analysis

Data were analyzed using IBM SPSS Statistics (version 28.0; IBM Corp., Armonk, NY, USA).

Descriptive statistics were reported as a mean with standard deviation (SD) or as absolute numbers with percentages (%) for normally distributed data. In cases of non-normally distributed data, the presentation of data were shown as median and interquartile range (IQR). Subgroup analyses were conducted using McNemar’s tests and ANOVA tests. Bonferroni’s correction for multiple testing was applied when appropriate. A *p*-value of <0.05 was considered statistically significant.

## 3. Results

A total of 539 women who visited the silicone outpatient clinic between December 2020 and December 2023 agreed to participate in this study and completed the baseline questionnaire. Participants were excluded from this analysis when they did not complete any of the follow-up questionnaires (*n* = 120) or if they had undergone implant removal before their initial clinic visit (*n* = 66). Ultimately, 353 women were included in the subsequent analysis, of which 182 (51.6%) had undergone explantation between their baseline and follow-up questionnaires. In cases where participants had completed multiple follow-up surveys, the last follow-up survey was used for analysis.

### 3.1. Baseline Characteristics

Baseline characteristics and implant details are shown in Table 1. Most women still had their first pair of implants (*n* = 240, 68.0%) at baseline, whereas 71 women had undergone implant replacement (20.1%). Notably, 42 women (11.9%) had already undergone three or more replacements of their implants. Fifty women (14.2%) had breast implants post-mastectomy following breast cancer. Reconstruction after bilateral preventive mastectomy in women with a BRCA-1, BRCA-2, CHEK-2 mutation, or another form of hereditary breast cancer was performed in 26 women (7.4%). The duration of implantation varied widely, ranging from 1 to 48 years from their implantation surgery to their first clinic visit, with a median of 12 years (IQR 8–20).

In 100 women (28.3%), a potential alternative diagnosis was identified that could partially or fully explain their symptoms. These alternative diagnoses encompassed conditions such as menopause, confirmed rheumatic diseases such as rheumatic arthritis, thyroid disorders, side effects from medication, and chronic conditions such as diabetes mellitus or chronic obstructive pulmonary disease (COPD).

Women were queried about previous visits to other medical specialists regarding their health issues. The majority of our cohort (275 women, 77.9%) disclosed having visited one or more medical specialists due to their systemic symptoms, while 124 women (35.1%) consulted with three or more specialists. The most frequently consulted specialists included plastic surgeons (173 women, 49.0%), internists (95 women, 26.9%), rheumatologists (92 women, 26.1%), neurologists (86 women, 24.4%), and cardiologists (49 women, 13.9%).

### 3.2. Baseline Survey: Reported Symptoms

Table 2 presents the systemic symptoms and their severity rating on a scale from 1 (negligible) to 10 (extremely severe). Notably, fatigue, sicca complaints, and arthralgia emerged as the most severe complaints. Additionally, the majority of women experienced one or more local symptoms attributed to their breasts (*n* = 294, 83.3%), among others breast pain or persistent pressure on the chest (Table 2). Additional local symptoms such as capsular contracture and deep itching in breasts and/or armpits were frequently mentioned in the free-text responses.

Moreover, all women were queried about other factors they believed might contribute to their specific symptoms. Notably, 31.2% of women attributed their fatigue in part to other causes, while in contrast, only 3.7% of women attributed their axillary lymphadenopathy to other factors (Table 2). In the free-text responses about factors contributing to their symptoms, answers varied widely. Frequently mentioned factors or diseases included osteoarthritis, menopause, stress, post-traumatic stress disorder (PTSD), fibromyalgia, rheumatoid arthritis, thyroid diseases, side effects of previous treatments with chemotherapy, or herniated cervical/lumbar discs (as an explanation for neuropathic symptoms).

On the basis of their symptom pattern and assessment of possible alternative diagnoses, the physician ultimately documented suspicion of BII as ‘high suspicion’ for 112 women (31.7%), ‘moderate suspicion’ for 147 women (41.6%), and ‘low suspicion’ for 94 women (26.6%).

### 3.3. Follow-Up Surveys

As early participants had the opportunity to complete up to three follow-up questionnaires, their follow-up periods extended up to three years. Conversely, patients who enrolled in 2023 had the opportunity to complete only one follow-up questionnaire (at 6 months). Consequently, the time from the first clinic visit until completion of the most recent follow-up survey ranged from 5–36 months, with a median of 12 months (IQR 8–24). At follow-up, 182 women (51.6%) had undergone surgical removal of their implants. Among them, 77 were categorized as having high suspicion of BII, 78 as moderate, while 27 women, despite being categorized as having low suspicion of BII, still chose to undergo explantation. The time from the explantation surgery to completing the follow-up questionnaire ranged from 1–18 months, with a median of 4.0 months (IQR 3.0–6.0). Importantly, there were no statistically significant differences between the explantation group and the non-explantation group, nor between the BII-suspicion groups, concerning baseline characteristics such as age, duration of implantation, reason for implantation, comorbidities, and history of intoxications.

When questioned about the reasons for explantation, many women identified the systemic symptoms as the main factor (40.5%), followed by local symptoms (22.2%), concerns about potential illness from silicone (17.9%), implant aging (10.3%), or implant rupture (8.3%).

### 3.4. Symptom Development Stratified by Varying Degrees of BII Suspicion

Table 3 illustrates the variations in symptom patterns among the three groups categorized by varying degrees of BII suspicion, encompassing both local and systemic symptoms.

Women exhibiting a high suspicion of BII reported significantly higher prevalence rates for all local symptoms, including breast pain (65 women, 58.0%), and persistent chest pressure (47 women, 42.0%), compared to the other two groups with moderate or low suspicion of BII. Additionally, we observed significant differences across all reported systemic symptoms. Women with a high suspicion of BII generally showed a greater baseline symptom burden compared to those with moderate or low suspicion of BII.

**Figure 1:**
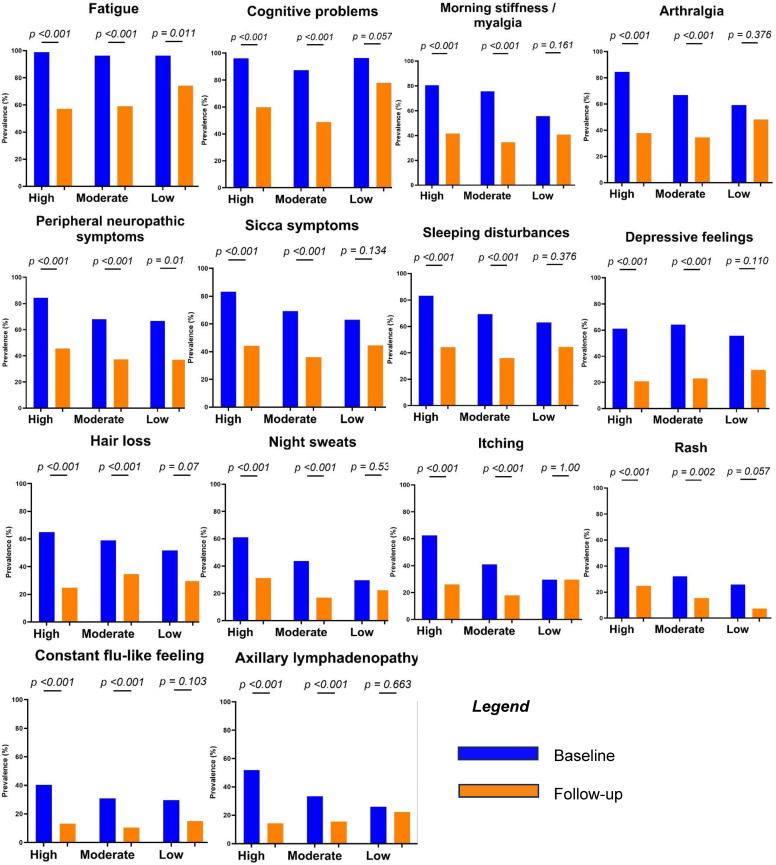
Symptom development after explantation per symptom, stratified by BII suspicion. Associated data can be found in Supplementary S2, Table S1.

Figure 1 (associated data can be found in Supplementary Table S1) presents the post-explantation symptom improvement per symptom, stratified by BII probability. The most substantial symptom improvement was observed among women with a high suspicion of BII, although noteworthy improvements were also observed in the group of women with moderate suspicion of BII. Conversely, women with a low suspicion of BII who still opted for implant removal displayed significant improvement only in fatigue and peripheral neuropathic symptoms (*p* = 0.011 and *p* = 0.018, respectively).

In addition, women at follow-up who did not opt for implant removal showed significant improvement in some of their symptoms as well (Supplementary Table S2).

### 3.5. Illness Perception and Impact on Daily Life

Table 4 presents the scores of the BIPQs stratified for BII suspicion. At baseline, the women with a high suspicion of BII reported a significant burden of their illness on their daily life, a higher sense of having no control over their illness, and expressed greater levels of worry compared to the women with a low suspicion of BII. Following explantation, women across all groups experienced a decrease in concern about their illness and a greater perceived sense of control. This difference was generally most pronounced in the group with the highest suspicion of BII, followed by the women with a moderate suspicion of BII.

Following explantation, 35% of women indicated no increase in insecurity regarding their aesthetics. However, 29.8% of women expressed feeling very insecure. Moreover, a substantial number of women (38.1%) stated feeling significantly less anxious after the surgery, although some women (22.2%) reported an increase in anxiety.

Importantly, a significant portion of women reported encountering challenges in their professional lives due to health issues. Specifically, 114 women (32.3%) reported either working fewer hours or discontinuing work altogether.

Lastly, to address potential selection bias, we assessed a group of 94 women who agreed to participate in their first clinic visit but did not complete any of the questionnaires. We found no significant differences in BII probability or implant details, except for their age, which was almost a decade younger than our study population. We do not anticipate that their completion of the questionnaires would have significantly altered our results.

## 4. Discussion

This prospective observational cohort study aimed to shed light on the clinical profile of women with silicone breast implants and varying degrees of BII suspicion. Women with a high or moderate suspicion of BII reported a consistent pattern of multiple typical systemic symptoms, including fatigue, cognitive symptoms, morning stiffness or myalgia, arthralgia, peripheral neuropathic symptoms, and sicca symptoms. Additionally, the vast majority of them reported local symptoms, most commonly breast pain, persistent pressure on the chest, and hypersensitivity of the breasts. Following explantation, there was a significant improvement in all systemic symptoms observed among women with a high or moderate suspicion of BII. However, among women with a low clinical suspicion of BII who opted for implant removal, improvements were limited to fatigue and peripheral neuropathic symptoms. This underscores the importance of clinical assessment by experienced physicians in discerning which women may benefit from explantation and which may not.

In line with our previous retrospective cohort study, the majority of women reported experiencing local symptoms, predominantly pain, persistent pressure on the chest, hypersensitive breasts, or axillary lymphadenopathy [9]. The prevalence of local symptoms was significantly higher among women with a high suspicion of BII in comparison to those categorized as moderate or low. Considering that the most substantial symptom improvement after explantation was observed in the group with a high BII suspicion, we believe that the presence of local symptoms alongside typical systemic symptoms serves as an important indicator of BII and, consequently, the efficacy of explantation. This observed interplay between both local and systemic symptoms lends support to the hypothesis implicating silicone-induced inflammation in symptom development of BII [4,25,26]. It is noteworthy that the group of women with a moderate suspicion of BII experienced a comparable degree of symptom improvement following explantation as the high suspicion group. This suggests that substantial symptom relief may be anticipated even in cases of moderate suspicion, emphasizing the importance of discussing the potential benefits of explantation also within this patient group.

Importantly, our study also documented the significant impact of health issues on the daily and professional lives of women with suspected BII. Many participants reported encountering challenges in their professional lives due to their health issues, with implications for work productivity, working hours, and overall quality of life. After explantation, women generally indicated feeling a greater sense of control over their illness, most pronounced by the women with a high suspicion of BII. While a significant proportion experienced substantially reduced anxiety levels following explantation, it is noteworthy that a small proportion reported feeling more anxious. This variation highlights the importance of considering individual psychological differences in response to explantation. In addition, our study illuminates the extensive medical consultations sought by this patient population and their associated financial burden. The majority of women (77.9%) had sought consultations with at least one medical specialist, and a substantial proportion (35.1%) sought consultations with three or more specialists, highlighting the multifaceted nature of their symptoms. One recently published study found similar results and even argued that medical specialist care utilization could serve as a relevant indicator of BII [23]. These findings underscore the significant impact of silicone breast-related symptoms on healthcare utilization and associated costs and highlight the broader societal implications of BII.

Currently, no (inter)national clinical guidelines are available for women with suspected BII. While several studies have proposed clinical directives, none of these guidelines were found to be directly applicable to the patient group we encounter at our clinic. However, reports of a typical symptom pattern seem consistent in several cohort studies [8,27,28,29,30]. The challenge with this pattern lies in the fact that many physicians argue that these symptoms are common in the general population, irrespective of breast implant status, citing examples such as fatigue and arthralgia. Nevertheless, our findings and expertise garnered at our silicone outpatient clinic suggest that when alternative diagnoses for these symptoms are meticulously excluded, particularly when the systemic symptoms are accompanied by local symptoms, a meaningful distinction can indeed be made. However, the ability to achieve this distinction requires not only expertise from the treating physicians but also considerable time for comprehensive medical assessment and supplementary testing. Additionally, it necessitates guiding the patient through an informed shared decision-making process regarding potential explantation and its associated consequences.

Interestingly, also the women from the non-explantation group demonstrated improvement of symptoms during follow-up, although not as pronounced as those who underwent explantation. In some cases, this improvement could be attributed to targeted treatment for alternative diagnoses that had been made. For others, merely the knowledge and reassurance that their symptoms were unrelated to the implants might have alleviated anxiety, thus reducing symptoms. This is shown by the moderate improvements in the BIPQ scores at follow-up we observed even among non-explantation cases. The role of psychological effects in influencing symptoms should not be overlooked in this context [31]. The nocebo effect, in which negative expectations lead to the perception of adverse effects, can influence how patients interpret their symptoms. Given the unknown etiology of BII, it is essential to balance seriously addressing patients concerns, as explantation significantly benefits those with a high suspicion of BII, while also considering the impact of psychological factors.

One of the strengths of our clinical study lies in its meticulous approach to data collection and analysis through our prospective design, ensuring the accuracy and reliability of the findings. Additionally, by focusing of the impact of these symptoms on daily life, we were able to provide a more comprehensive understanding of the challenges faced by affected individuals.

Nevertheless, our study had its limitations. Selection bias may have influenced our results, as participants who chose to enroll in the study may have differed from those who did not complete their questionnaires. However, our subgroup analysis of women who agreed to participate but did not complete questionnaires showed only an age difference, which we do not believe significantly altered our results. Furthermore, no randomization was employed, as the decision to undergo explantation was left to the discretion of the women involved. Moreover, recall bias is inherent in every study utilizing patient-reported data, although efforts were made to minimize this through the free-text responses in the questionnaire. Lastly, the relatively short follow-up time of 4 [range 1–18] months may have limited our ability in some patients to assess long-term symptom improvement. Some women may still be in the recovering phase up to a year after their surgery, potentially leading to underreporting of symptom improvement. Conversely, one could argue that the improvement in symptoms may have been caused by a placebo effect from the surgery, due to the short follow-up time [32]. However, in our previous retrospective study, we observed that the improvement was enduring with a median follow-up time of more than 3 years [9].

Unfortunately, the short follow-up time from our current study did limit our ability to reliably assess local symptoms after explantation. Many women indicated experiencing ongoing local symptoms attributed to the healing process following explantation surgery, or due to additional reconstructive surgeries. However, in our previous retrospective study, we observed a significant reduction in local symptoms following explantation as well [9]. As we intend to continue these questionnaires for the following years, we hope to further explore the development of local symptoms over time.

Considering the improvements in both symptoms and illness perception observed within the groups of women with high and moderate suspicion of BII, the time has come to offer thorough medical evaluation and treatment to this patient population. It is essential for medical professionals to acknowledge the validity of these symptoms and to take appropriate action. Importantly, we observed symptom improvement across all patients, either due to effective treatment of the underlying cause or simply due to recognition of the patient and her symptoms.

We recommend that clinicians carefully consider the possibility of silicone breast implant-related illness when encountering patients presenting with a pattern of both local and systemic symptoms, particularly after excluding other plausible causes. In such cases, implant removal should be considered. Nevertheless, personalized assessment and advice remain pivotal, as not all individuals experience improvement after explantation, with some patients even reporting feeling more anxious after the surgery.

## 5. Conclusions

By sharing our results and experiences, we aim to provide guidance in the diagnostic trajectory of women with silicone breast implants and suspected BII. We observed a typical pattern of multiple systemic symptoms among women with a high suspicion of BII, by which the accompanied presence of local symptoms can serve as a relevant indicator of BII. All symptoms decreased significantly after explantation in women with a high or moderate suspicion of BII, in contrast to the women with a low suspicion of BII. The symptom improvement was accompanied by a decrease in anxiety and a greater sense of control over their illness. Experienced physicians play a crucial role in assessing and managing this patient group effectively. Moreover, addressing the psychological and financial burdens associated with BII is imperative to ensure optimal patient care and healthcare system sustainability. These findings emphasize the need to develop effective clinical approaches to help this patient population.

## Figures and Tables

**Table 1:** Baseline characteristics.

Characteristics	Patients at Baseline (*n* = 353)
Age, in years	48.0 (SD 11.7)
Reason for implantation	
Cosmetic	277 (78.5%)
Reconstruction	76 (21.5%)
Implant type	
Silicone gel-filled implants	319 (90.4%)
Saline or hydrogel implants	5 (1.4%)
Unknown	29 (8.2%)
Implantation duration at clinic visit, in years	12.0 [8.0–20.0]
Implant rupture current implants *	54 (15.3%)
Implant brand	
Allergan	108 (30.6%)
Mentor	49 (13.9%)
Eurosilicone	44 (12.5%)
Other	52 (14.7)
Unknown	100 (28.3%)
Clinical suspicion of BII	
High	112 (31.7%)
Moderate	147 (41.6%)
Low	94 (26.6%)
Intoxications	
Active smoker	90 (19.8%)
Alcohol	158 (44.8%)
Drugs	8 (2.3%)
Medical comorbidities	
History of breast cancer	50 (14.2%)
Chemotherapy	21 (5.9%)
Auto-immune diseases Includes auto-immune thyroid disease (34), RA (6), IBD (6), M. Sjögren (5), SpA (5), SLE (4), psoriasis (4), sarcoidosis (3), APS (3), SSc (2), PMR (2)	83 (23.5%)
Fibromyalgia	47 (13.3%)
Raynaud’s phenomenon	9 (2.5%)
ME/CFS	6 (1.7%)
Irritable Bowel Syndrome (IBS)	27 (7.6%)
Depression/burn out	55 (15.6%)
Migraine	23 (6.5%)

Data shown are number (percentage) or mean (SD). APS: Antiphospholipid Syndrome, ME/CFS: Myalgic Encephalomyelitis/Chronic Fatigue Syndrome, IBD: Inflammatory Bowel Disease, PMR: Polymyalgia Rheumatica, RA: Rheumatoid Arthritis, SLE: Systemic Lupus Erythematodus, SpA: Spondyloarthritis, SSc: Systemic Sclerosis. * implant ruptures were diagnosed through ultrasound/MRI or during operation.

**Table 2:** Reported systemic and local symptoms at the time of the first clinic visit.

	Reported Symptoms First Clinic Visit (*n* = 353)	Severity Rating (1–10)	Attribution to Other Factors *
Fatigue	324 (91.8%)	7.4	110 (34.0%)
Cognitive problems (memory loss, loss of concentration)	312 (88.4%)	6.3	83 (26.6%)
Morning stiffness/myalgia	248 (70.3%)	6.4	73 (29.4%)
Arthralgia	242 (68.6%)	7.0	73 (29.4%)
Peripheral neuropathic symptoms (tingling, neuralgia)	235 (66.6%)	6.6	41 (17.4%)
Sicca (dry eyes, dry mouth)	233 (66.0%)	7.2	36 (15.4%)
Sleeping disturbances	220 (62.3%)	6.3	48 (21.8%)
Depressive feelings	191 (54.1%)	6.3	46 (24.0%)
Hair loss	182 (51.6%)	5.8	34 (18.7%)
Night sweats	150 (42.5%)	6.2	44 (29.3%)
Itching	144 (40.8%)	5.8	14 (9.7%)
Rash	119 (33.7%)	5.4	23 (19.3%)
Constant flu-like feeling	100 (28.3%)	6.5	15 (15%)
Axillary lymphadenopathy	108 (30.6%)	5.3	4 (3.7%)
Breast pain	150 (42.5%)	n.a.	n.a.
Persistent pressure on chest	112 (31.7%)	n.a.	n.a.
Hypersensitivity breast(s)	98 (27.8%)	n.a.	n.a.
Painful lumps breasts/armpits	81 (22.9%)	n.a.	n.a.

Additional severity score per systemic symptom, ranging from 1 (minimal severity) to 10 (reflecting maximal severity). An additional column with numbers and percentages of women who believe their symptoms might be attributed to other factors. Data are shown as numbers (n) and percentages (%). n.a.: not applicable. * interpretation of patients themselves.

**Table 3:** Reported systemic and local symptoms at baseline, stratified for women with varying degrees of BII suspicion.

	Women with High BII Suspicion (*n* = 112)	Women with Moderate BII Suspicion (*n* = 147)	Women with Low BII Suspicion (*n* = 94)	ANOVA Test *p*-Value (95%-CI) *
Fatigue	108 (96.4%)	138 (93.9%)	78 (83.0%)	0.01 (0.23–0.04)
Cognitive problems (memory loss, loss of concentration)	108 (96.4%)	125 (85.0%)	68 (72.3%)	<0.001 (0.13–0.36)
Morning stiffness/myalgia	93 (83.0%)	110 (74.8%)	45 (47.9%)	<0.001 (0.50–0.20)
Arthralgia	95 (84.8%)	104 (70.7%)	43 (45.7%)	<0.001 (0.54–0.24)
Peripheral neuropathic symptoms (tingling, neuralgia)	95 (84.8%)	95 (64.6%)	45 (47.9%)	<0.001 (0.22–0.56)
Sicca (dry eyes, dry mouth)	91 (81.3%)	97 (66.0%)	45 (47.9%)	<0.001 (0.18–0.49)
Sleeping disturbances	89 (79.5%)	94 (63.9%)	37 (39.4%)	<0.001 (0.25–0.56)
Depressive feelings	66 (58.9%)	89 (60.5%)	36 (38.3%)	0.002 (0.07–0.38)
Hair loss	67 (59.8%)	75 (51.0%)	40 (42.6%)	0.041 (0.01–0.34)
Night sweats	63 (56.3%)	65 (44.2%)	22 (23.4%)	<0.001 (0.49–0.17)
Itching	63 (56.3%)	59 (40.1%)	22 (23.4%)	<0.001 (0.17–0.49)
Rash	52 (46.6%)	47 (32.0%)	20 (21.3%)	<0.001 (0.10–0.41)
Constant flu-like feeling	44 (39.3%)	41 (27.9%)	15 (16.0%)	<0.001 (0.08–0.38)
Axillary lymphadenopathy	50 (44.6%)	44 (29.9%)	14 (14.9%)	<0.001 (0.45–0.15)
Breast pain	65 (58.0%)	65 (44.2%)	20 (21.3%)	<0.001 (0.21–0.53)
Persistent pressure on chest	47 (42.0%)	50 (34.0%)	15 (16.0%)	<0.001 (0.11–0.41)
Hypersensitivity breast(s)	45 (40.2%)	39 (26.5%)	14 (14.9%)	<0.001 (0.11–0.40)
Painful lumps breasts/armpits	35 (31.3%)	33 (22.4%)	13 (13.8%)	0.009 (0.03–0.31)

* ANOVA tests with Bonferroni correction for multiple testing (*p*-value and 95%-CI). *p*-values represent the largest difference between 2 of the 3 groups. Data are shown as numbers (n) and percentages (%).

**Table 4:** Rating of the Brief Illness Perception Questionnaire (BIPQ), cross-cultural adapted Dutch version (IPQ-K) [24] at baseline and after explantation.

		Baseline		Difference in BIPQ Score after Explantation		
	Women with High BII Suspicion (*n* = 112)	Women with Moderate BII Suspicion (*n* = 147)	Women with Low BII Suspicion (*n* = 94)	Women with High BII Suspicion (*n* = 77)	Women with Moderate BII Suspicion (*n* = 78)	Women with Low BII Suspicion (*n* = 27)
1. Influence of illness on daily life (consequences)	7.9	7.5	6.0	−2.8 *	−2.1	−1.8
2. Length of illness on life (timeline)	7.6	7.8	7.2	−1.7	−1.3	−0.4
3. Feeling of control of illness (personal control)	7.4	7.5	6.4	−1.3	−1.5	−0.5
4. Feeling that current treatment will help for illness (treatment control)	4.2	4.5	5.0	+1.2	+1.0	+0.2
5. Severity experiencing illness (identity)	7.9	7.6	6.2	−2.4	−2.3	−1.2
6. Worried about illness (concern)	8.3	7.3	6.1	−3.1	−3.1	−2.0
7. Understanding illness (understanding)	5.2	5.2	5.1	−0.1	−1.3	−0.8
8. Influence illness on mood (emotional response)	7.6	6.7	5.8	−2.6	−2.0	−1.7

* Indicates the difference in BIPQ scores between baseline and post-explantation within the explantation group, stratified by BII suspicion (N = 77, N = 78, and N = 27 at baseline, respectively). Scores are measured on a severity scale from 1 to 10, with the scores indicating: question 1: no influence on daily life (1)–very much influence on daily life (10); question 2: short duration of illness (1)–lifelong duration of illness (10); question 3: having much control of illness (1)–having no control of illness (10); question 4: feeling that treatment has helped (1)–feeling that treatment will not help (10); question 5: experiencing no symptoms (1)–experiencing many symptoms (10); question 6: no concern about illness (1)–a lot of concern about illness (10); question 7: fully understanding illness (1)–no understanding of illness (10); question 8: no influence of illness on mood (1)–a lot of influence of illness on mood (10).

## Data Availability

Data will be made available on request.

## Supplementary Materials

The following supporting information can be downloaded at: https://www.mdpi.com/article/10.3390/jcm13154394/s1, Supplementary S1: Guideline; Supplementary S2: Table S1. Reported systemic symptoms among women after explantation, stratified by suspected probability of Breast Implant Illness. Table S2. Reported systemic symptoms before and after explantation, stratified for the explantation and non-explantation group. References [5,8,9,10,11,15,17,19,33,34,35,36,37,38,39,40,41,42] are cited in the supplementary materials.
