# Supplementary material for: A Prospective Observational Clinical Cohort of Women with Suspected Breast Implant Illness
Source: J Clin Med. 2024 Jul 27;13(15):4394. doi: 10.3390/jcm13154394 (PMC11313056; doi:10.3390/jcm13154394)
Supplement: Supplementary file 1 [file jcm-13-04394-s001.zip › jcm-3077897-supplementary.pdf]

Clinical protocol for the specialized silicone outpatient clinic of Amsterdam UMC, VUmc location

Department of Internal Medicine

English version 1, April 2024

Background

Since 2011, the specialized Silicone Outpatient Clinic at Amsterdam UMC, VU University Medical Center, has been evaluating women with silicone breast implants who present with systemic symptoms. The clinic aims to determine whether these symptoms may be linked to their silicone breast implants. Despite the absence of official (inter)national guidelines for this patient population, several studies have highlighted a distinct pattern of symptoms observed in some women with breast implants.[5, 8] While the precise pathophysiology driving these symptoms remains unclear, empirical evidence consistently demonstrates symptom improvement in approximately two-thirds of women who undergo implant removal (explantation).[10, 11] One prevailing hypothesis suggests that the presence of implants may trigger a low-grade inflammatory response, contributing to symptom manifestation. Given the often prolonged duration of these symptoms, their impact on patients' daily lives and employability can be substantial.

Benefitting from the longstanding existence of the Silicone Outpatient Clinic and its extensive follow-up program, Amsterdam UMC currently has a unique and, to the best of our knowledge, the largest patient population worldwide in this field. Drawing upon both scientific research and quality assessments conducted at the clinic, alongside accumulated clinical expertise, we present the following recommendations regarding diagnostic approaches at the internal medicine outpatient clinic for these patients.

Explanation to Patients

Many women with suspected silicone-associated systemic symptoms often feel their concerns are dismissed by their healthcare providers. Furthermore, media coverage contributes to fears regarding silicone migration and potential illness among these women. Addressing these concerns is pivotal.

Key points/Information:

- Not all individuals with silicone breast implants develop symptoms, even in the case of implant rupture.
- The precise etiology of these symptoms remains unclear. It is hypothesized that they may arise from an inflammatory response or allergic reaction, with variations in individual host responses. However, a toxicological origin cannot be definitively ruled out.
- There is currently no standardized test available to confirm or exclude silicone-associated symptoms. Our clinic's research indicates that 2 out of 3 women exhibiting a characteristic symptom pattern experience symptom improvement following explantation, although 30% do not - percentages consistent with existing literature. [9]

- All silicone breast implants exhibit a phenomenon known as 'gel bleed', wherein silicone particles migrate into distant tissues within the body. These migrated silicone depositions have the potential to induce cause inflammation, with axillary lymph nodes being the most commonly affected site. To date, there is no method known to effectively remove distant silicone particles from the body.
- The concentration of silicone particles in the bloodstream cannot be quantified.
- A hair test (measuring platinum) does not provide conclusive evidence of implant leakage. [33]
- Saline-filled implants feature a silicone shell that may degrade or rupture over time. Some individuals with saline implants also report experiencing symptoms.
- Silicone breast implants have been associated with an increased risk of a rare subtype of non-Hodgkin lymphoma known as BIA-ALCL (breast implant-associated anaplastic large cell lymphoma). These cases have primarily, though not exclusively, been associated with Biocell macro-textured and polyurethane implants. These particular implants have been discontinued since 2018 (see section 'BIA-ALCL').
- Additionally, various types of silicone breast implants have been associated with squamous cell carcinomas and other forms of lymphomas within the capsule surrounding the implant (See section 'BIA-SCC'). [34]
- All silicone implants undergo degradation over time and have a finite lifespan: the longer they remain implanted, the higher the risk of rupture. According to the literature, the 10-year rupture rate for cosmetic implants is approximately 10-15%, while for reconstructive implants, it ranges from 20-25%. [15,17] It is up to the patient to consider the replacement or removal of implants after 10-15 years to mitigate the risk of ruptures and silicone leakage, considering factors such as costs, surgical risks, and evidence of wear observed on imaging. In cases where silicone-associated symptoms are suspected, it is advisable to opt for explantation rather than replacement.

Medical History

<u>Characteristics of Implants</u>

- When were the implants initially placed? Have they ever undergone replacement? If so, how frequently, when, and were there any instances of rupture? What is the age of the current implants?
- What was the indication or the initial placement? (Cosmetic augmentation, reconstruction following mastectomy due to breast cancer or mastopathy, prophylactic mastectomy for BRCA1/2 mutation, etc.)
- What is the brand and type of implants?

  Pay attention to the following brands/types:
  - Implants from the PIP brand (also known as Rofil/M-implants) have been recalled due to the use of industrial silicone instead of medical silicone and have been prohibited since 2010. Research has indicated these implants have an increased tendency to rupture. [35,36]
  - 'Biocell' implants (brand Allergan/Inamed/McGhan) and polyurethaan implants (Silimed) have been associated with an increased risk of BIA-ALCL and have not been used since 2018. [37]

Assessment of common systemic silicone-associated symptoms

In the majority of patients, we observe a combination of systemic and local symptoms. Specifically, around 75% of patients report having both local symptoms and multiple systemic symptoms. [9]

- (Severe) fatigue
- Arthralgias
- Morning stiffness
- Myalgias
- Nights sweats
- Tingling/numbness in hands (sometimes induced by palpation of axillary lymph nodes)
- Sicca symptoms
- Persistent 'flu-like' sensation
- Shortness of breath with persistent tightness around the chest
- Cognitive symptoms, such as memory problems or 'brain fog'
- Itchy skin rash or generalized itching without rash
- Hair loss (both diffuse and bald patches on the scalp)
- Palpitations
- Increase in pre-existing or development of new allergies/intolerances after implant insertion

Assessment of common local silicone-associated symptoms

- Breast symptoms (pain, stabbing sensations, intense deep itching)
- Axillary symptoms (pain, swelling, itching)
- Axillary lymphadenopathy (painful, swollen nodes upon palpation)
- Radiating pain, tingling, or weakness in arms/hands
- Inability to lie on breasts
- Thoracic pain

Inquiring about other potential causes of symptoms

- Comprehensive medical history to rule out alternative diagnoses
  (considerations include: Rheumatic diseases, autoimmune disorders, thyroid disorders, menopausal symptoms, medication side effects, past treatments (chemotherapy), psychosocial history, malignancies (B-symptoms), intoxications, pulmonary complaints, and cardiac symptoms.)
- Family history: autoimmune disorders, rheumatic diseases, malignancies.

Breast implant-associated anaplastic large cell lymphoma (BIA-ALCL)

As previously discussed, various brands and types of breast implants are associated with an elevated risk of BIA-ALCL. This risk varies considerably, with estimates ranging from 1:2000 to 1:70.000. A Dutch study estimated the risk to be 1:7000 women by the age of 75, while more recent research suggested a cumulative risk of 1:100 over 20 years in reconstructive patients with Biocell implants. [38,39]

Early manifestations of BIA-ALCL typically include sudden unilateral swelling (seroma formation), erythema of the breast, ulcerations, or nodules in the breast or axilla. These symptoms may be accompanied by constitutional symptoms such as weight loss, fever, and night sweats. Fortunately, BIA-ALCL generally responds well to treatment, with a mortality rate estimated at 2.8% after one year.

Breast implant-associated squamous cell carcinoma (BIA-SCC)

On September 8, 2022, the FDA issued an update regarding reports of extremely rare, but aggressive squamous cell carcinomas (SCCs) and other types of lymphomas distinct from BIA-ALCL occurring within the capsules surrounding silicone breast implants. To date, 19 cases have been documented in women with various types of implants (smooth, textured, saline, and silicone), and in both cosmetic and reconstructive patients. The clinical presentation resembles that of BIA-ALCL, although capsule formation is more prevalent. Notably, there is a high reported mortality rate of 43.8% at 6 months. [40]

Physical Examination

General physical examination (height, weight, vital signs, cardiac/pulmonary examination, joints, lymph node stations). Neurological examination should be performed if indicated.

Specific:

<u>Breasts:</u> Breast symmetry? Retractions upon changes in position? Nipple abnormalities? Skin abnormalities? Palpable indurations/siliconomas? Baker grading?

*(**Baker** 1: no palpable capsule, the breast feels as soft as an unoperated breast; **Baker 2**: minimal firmness, the breast is less soft, and the breast prosthesis is palpable but not visible; **Baker 3**: moderate firmness; the breast is harder, the prosthesis is easily palpable and visible (or deformed); **Baker 4**: severe contracture, the breast is hard, painful, and cold; deformation is often clearly visible)*

<u>Axillae:</u> palpable enlarged lymph nodes? Are they painful upon palpation?

Additional follow-up

- **Blood tests aimed at excluding other causes or the symptoms, including:**
  - Erythrocyte sedimentation rate (ESR), C-reactive protein (CRP)
  - Hemoglobin (Hb), leukocytes, platelets, leukocyte differential count
  - Liver and renal function tests, ferritin level
  - Vitamin D and B12 levels
  - Thyroid function tests
  - Antinuclear antibody (ANA); if positive, extractable nuclear antigen (ENA) testing
  - *Further tests as deemed necessary by the physician, including rheumatological tests, such as cyclic citrullinated peptide (CCP) antibodies/rheumatoid factor tests, etc.*
- **Radiological assessment**
  Prompt referral for radiological evaluation to exclude silicone leakage, such as ultrasound or MRI, if not recently performed elsewhere. We recommend initiating with a breast and axillary ultrasound (see below). If inconclusive, consider proceeding with a breast MRI following a silicone protocol.
- **Suspicion of BIA-ALCL or other malignancies**
  In case of suspicion of BIA-ALCL or BIA-SCC, immediately request for breast ultrasound and cytological aspiration.

<u>Ultrasound</u>

Ultrasound is a straightforward, non-invasive, and cost-effective diagnostic tool that can provide valuable information in cases of silicone-associated symptoms. The sensitivity of ultrasound in detecting implant ruptures is approximately 80%. [41] While MRI has a higher sensitivity in detecting implant ruptures, ultrasound of the axillary lymph nodes is more sensitive in identifying silicone depositions in the axillary lymph nodes (termed the 'snowstorm' phenomenon). Therefore, it is advisable to request both breast and axillary ultrasound examinations. Our research at Amsterdam UMC indicates that the sensitivity and specificity of ultrasound in detecting implant ruptures are

nearly equivalent to MRI, whereas MRI's sensitivity in identifying silicone deposits in the axillary lymph nodes is only 44% compared to ultrasound.

MRI

Breast MRI of the breast implants is ideally performed following a specialized silicone protocol, enabling a thorough assessment of implants and potential silicone leakage. This protocol enhances the sensitivity of MRI in detecting implant ruptures, achieving a diagnostic accuracy of over 90% certainty. [15]

Note: it is important to note that conventional breast tissue assessment is limited with this silicone-specific protocol, precluding the diagnosis of tissue abnormalities like suspected breast carcinoma.

Indications for requesting radiological assessment include:

- Asymmetry or alterations in breast shape;
- Breast swelling (start with ultrasound to allow for possible needle aspiration)
- Recent onset of pain in one or both breasts
- Axillary pain or discomfort
- Implants aged >10 years
- Extreme anxiety in the patient, even in the absence of specific symptoms

Note: mammography is not suitable for a thorough evaluation of breast implants

Diagnosis

The consideration of 'silicone breast implant-related symptoms' or 'breast implant illness' arises only after the exclusion of alternative diagnoses (diagnosis per exclusionem). In cases of abnormal or inexplicable findings, or when an alternative diagnosis is suspected, consultation with a specialist is advised if deemed necessary.

When a patient presents with a multitude of typical physical symptoms and other potential diagnoses that have been thoroughly investigated and eliminated, a significant suspicion of silicone-associated symptoms emerges. Notably, this suspicion persists even when radiological examinations indicate intact implants.

In such cases, it is recommended to engage in a detailed discussion with the patient regarding the possibility of implant removal. Implant removal rather than replacement is preferred, given the potential recurrence of symptoms associated with new implants.

If doubt exists regarding the diagnosis, whether from the physician or the patient, a decision to wait may be made. However, if new symptoms develop or existing ones worsen, indicative of silicone-associated symptoms, proceeding with implant removal may be advisable.

In cases where few or no typical symptoms are present, there is a low suspicion of silicone-associated symptoms, and implant removal is not warranted.

PM. Instruct patients to contact their general practitioner if there is an increase in symptoms and/or development of new symptoms that are not typical for silicone-associated symptoms, to exclude other causes.

Follow-up

Symptom improvement after implant removal may vary in timing, with some experiencing immediate relief, while others may require several months. Our clinic's research indicates that optimal improvement occurs in women who undergo implant removal within 10 years of initial placement and symptom onset. Conversely, those with confirmed silicone deposits appear to have less favorable outcomes.

Reassessment of symptoms at the 6-month mark post-removal is crucial, especially if patients report no improvement. Early reassessment is warranted if symptoms worsen or new ones arise. Repeating (physical) examination and blood tests is essential, along with considering alternative conditions.
In a subset proportion of our patients who do not improve after explantation, we have observed positive responses to a short course of prednisone treatment, provided other potential causes are ruled out. Furthermore, some patients benefit from participation in a rehabilitation program.

Axillary silicone depositions

The potential long-term health implications of silicone deposits within axillary lymph nodes remain uncertain. Surgical excision of lymph nodes containing silicone is generally not recommended due to associated risks, unless these nodes elicit significant local pain or malignancy is suspected. To date, there has only been one documented case worldwide of a woman developing BIA-ALCL in an axillary node containing silicone, occurring two years after implant removal. [42]
Nevertheless, the possibility remains that silicone leakage into lymph nodes could perpetuate persistent systemic symptoms. In several cases of substantial silicone leakage and persistent symptoms after explantation, PET-avid lymph nodes corresponding to silicone-containing nodes, including axillary, parasternal, and even cervical nodes were observed on PET-CT scans. This suggests the persistence of an inflammatory response despite explantation in instances of such silicone leakage. In one patient from our clinic, who had a history of implant rupture and is currently suffering from inflammatory sclerosing inflammation of the thighs, silicone particles were identified in the revised biopsies of the inflamed tissue. This indicates that in rare cases, individual host responses to the silicone particles may be severe. [19]

Table S1. Reported systemic symptoms among women after explantation, stratified by suspected probability of Breast Implant Illness.

	**Women with high BII suspicion at baseline (n=77)**	**Women with high BII suspicion after explantation (n=77)**	**Women with moderate suspicion of BII at baseline (n=78)**	**Women with moderate suspicion of BII after explantation (n=78)**	**Women with low BII suspicion at baseline (n=27)**	**Women with low BII suspicion after explantation (n=27)**
**Fatigue**	76 (98.7%)	44 (57.1%) *p <0.001*	75 (96.2%)	46 (59.0%) *p <0.001*	26 (96.3%)	20 (74.1%) *p = 0.011*
**Cognitive problems (memory loss, loss of concentration)**	74 (96.1%)	46 (59.7%) *p <0.001*	68 (87.2%)	38 (48.7%) *p <0.001*	26 (96.3%)	21 (77.8%) *p = 0.057*
**Morning stiffness/myalgia**	62 (80.5%)	32 (41.6%) *p <0.001*	59 (75.6%)	27 (34.6%) *p = <0.001*	15 (55.6%)	11 (40.7%) *p = 0.161*
**Arthralgia**	65 (84.4%)	29 (37.7%) *p <0.001*	52 (66.7%)	27 (34.6%) *p <0.001*	16 (59.3%)	13 (48.1%) *p = 0.376*

**Peripheral neuropathic symptoms (tingling, neuralgia)**	65 (84.4%)	35 (45.5%) *p* <0.001	53 (67.9%)	29 (37.2%) *p* <0.001	18 (66.7%)	10 (37.0%) *p* = 0.018
**Sicca (dry eyes, dry mouth)**	64 (83.1%)	34 (44.2%) *p* <0.001	54 (69.2%)	28 (35.9%) *p* <0.001	17 (63.0%)	12 (44.4%) *p* = 0.134
**Sleeping disturbances**	63 (81.8%)	34 (44.2%) *p* <0.001	47 (60.3%)	25 (32.1%) *p* <0.001	15 (55.6%)	12 (44.4%) *p* = 0.376
**Depressive feelings**	47 (61.0%)	16 (20.8%) *p* <0.001	50 (64.1%)	18 (23.1%) *p* <0.001	14 (55.6%)	8 (29.6%) *p* = 0.110
**Hair loss**	50 (64.9%)	19 (24.7%) *p* <0.001	46 (59.0%)	27 (34.6%) *p* <0.001	15 (55.6%)	8 (29.6%) *p* = 0.070
**Night sweats**	47 (61.0%)	24 (31.2%) *p* <0.001	34 (43.6%)	13 (16.7%) *p* <0.001	8 (29.6%)	6 (22.2%) *p* = 0.537
**Itching**	48 (62.3%)	20 (26.0%) *p* <0.001	32 (41.0%)	14 (17.9%) *p* = 0.002	8 (29.6%)	8 (29.6%) *p* = 1.00
**Rash**	42 (54.5%)	19 (24.7%) *p* <0.001	25 (32.1%)	12 (15.4%) *p* = 0.004	7 (25.9%)	2 (7.4%) *p* = 0.057
**Constant flu-like feeling**	31 (40.3%)	10 (13%) *p* <0.001	24 (30.8%)	8 (10.3%) *p* <0.001	8 (29.6%)	4 (14.8%) *p* = 0.103
**Axillary lymphadenopathy**	43 (51.9%)	11 (14.3%) *p* <0.001	26 (33.3%)	12 (15.4%) *p* = 0.001	7 (25.9%)	6 (22.2%) *p* = 0.663

Additional McNemar tests between baseline and follow-up symptoms prevalence per group (*p*-value). Data are shown as numbers (n) and percentages (%).

Table S2. Reported systemic symptoms before and after explantation, stratified for the explantation and non-explantation group.

	**Explantation group at baseline (n=182)**	**Explantation group at follow up* (n=182)**	***p*-value**	**Non-explantation group baseline (n=171)**	**Non-explantation group follow-up (n=171)**	***p*-value**
**Fatigue**	177 (97.3%)	110 (60.4%)	<0.001	147 (86.0%)	136 (79.5%)	0.048
**Cognitive problems (memory loss, loss of concentration)**	168 (92.3%)	105 (57.7%)	<0.001	133 (77.8%)	123 (71.9%)	0.114

**Morning stiffness/myalgia**	136 (74.7%)	70 (38.5%)	<0.001	112 (65.6%)	86 (50.3%)	<0.001
**Arthralgia**	133 (73.1%)	69 (37.9%)	<0.001	109 (63.7%)	93 (54.4%)	0.032
**Peripheral neuropathic symptoms (tingling, neuralgia)**	136 (74.7%)	74 (40.7%)	<0.001	99 (57.9%)	79 (46.2%)	0.011
**Sicca (dry eyes, dry mouth)**	135 (74.2%)	74 (40.7%)	<0.001	98 (57.3%)	75 (43.9%)	0.002
**Sleeping disturbances**	125 (68.7%)	71 (39.0%)	<0.001	95 (55.6%)	85 (49.7%)	0.132
**Depressive feelings**	111 (61.0%)	42 (23.1%)	<0.001	80 (46.8%)	63 (36.8%)	0.021
**Hair loss**	111 (61.0%)	54 (29.7%)	<0.001	71 (41.5%)	53 (31.0%)	0.008
**Night sweats**	89 (48.9%)	43 (23.6%)	<0.001	61 (35.7%)	43 (25.1%)	0.006
**Itching**	88 (48.4%)	42 (23.1%)	<0.001	56 (32.7%)	40 (23.4%)	0.018
**Rash**	74 (40.7%)	33 (18.1%)	<0.001	45 (26.3%)	26 (15.2%)	<0.001
**Constant flu-like feeling**	63 (34.6%)	22 (12.1%)	<0.001	37 (21.6%)	27 (15.8%)	0.086
**Axillary lymphadenopathy**	74 (40.1%)	29 (15.9%)	<0.001	35 (20.5%)	34 (19.9%)	0.866

Additional columns with severity score per symptom, before and after explantation. Data are shown as numbers (n) and percentages (%). McNemar tests were performed to statistically test the difference between baseline and follow-up, in the two groups. * median follow-up time from surgery to questionnaire was 4 months [range 1-18].
